# Vasculitogenic T Cells in Large Vessel Vasculitis

**DOI:** 10.3389/fimmu.2022.923582

**Published:** 2022-06-15

**Authors:** Ryu Watanabe, Motomu Hashimoto

**Affiliations:** Department of Clinical Immunology, Osaka Metropolitan University Graduate School of Medicine, Osaka, Japan

**Keywords:** CD4+ T cells, CD8+ T cells, giant cell arteritis, regulatory T cells, Takayasu arteritis

## Abstract

Vasculitis is an autoimmune disease of unknown etiology that causes inflammation of the blood vessels. Large vessel vasculitis is classified as either giant cell arteritis (GCA), which occurs exclusively in the elderly, or Takayasu arteritis (TAK), which mainly affects young women. Various cell types are involved in the pathogenesis of large vessel vasculitis. Among these, dendritic cells located between the adventitia and the media initiate the inflammatory cascade as antigen-presenting cells, followed by activation of macrophages and T cells contributing to vessel wall destruction. In both diseases, naive CD4^+^ T cells are polarized to differentiate into Th1 or Th17 cells, whereas differentiation into regulatory T cells, which suppress vascular inflammation, is inhibited. Skewed T cell differentiation is the result of aberrant intracellular signaling, such as the mechanistic target of rapamycin (mTOR) or the Janus kinase signal transducer and activator of transcription (JAK-STAT) pathways. It has also become clear that tissue niches in the vasculature fuel activated T cells and maintain tissue-resident memory T cells. In this review, we outline the most recent understanding of the pathophysiology of large vessel vasculitis. Then, we provide a summary of skewed T cell differentiation in the vasculature and peripheral blood. Finally, new therapeutic strategies for correcting skewed T cell differentiation as well as aberrant intracellular signaling are discussed.

## Introduction

Vasculitis is an autoimmune disorder that causes inflammation of blood vessels and multiple organ damage. Large vessel vasculitis (LVV) primarily affects the aorta and its major branches and can be divided into two disease categories: giant cell arteritis (GCA) and Takayasu’s arteritis (TAK) (1). GCA is common in individuals over 50 years of age, especially in their 60s to 80s (2). Symptoms related to GCA include fever, headache, jaw claudication, and visual disturbances (3). Polymyalgia rheumatica is often accompanied by extravascular manifestations (4). In contrast, TAK is common in patients under 50 years of age, especially in Asian women in their 20s to 40s (5). TAK results in fever, general malaise, pulselessness, renovascular hypertension, and aortic regurgitation (6). Ulcerative colitis and erythema nodosum are well-known extravascular manifestations (7, 8).

**Figure 1:**
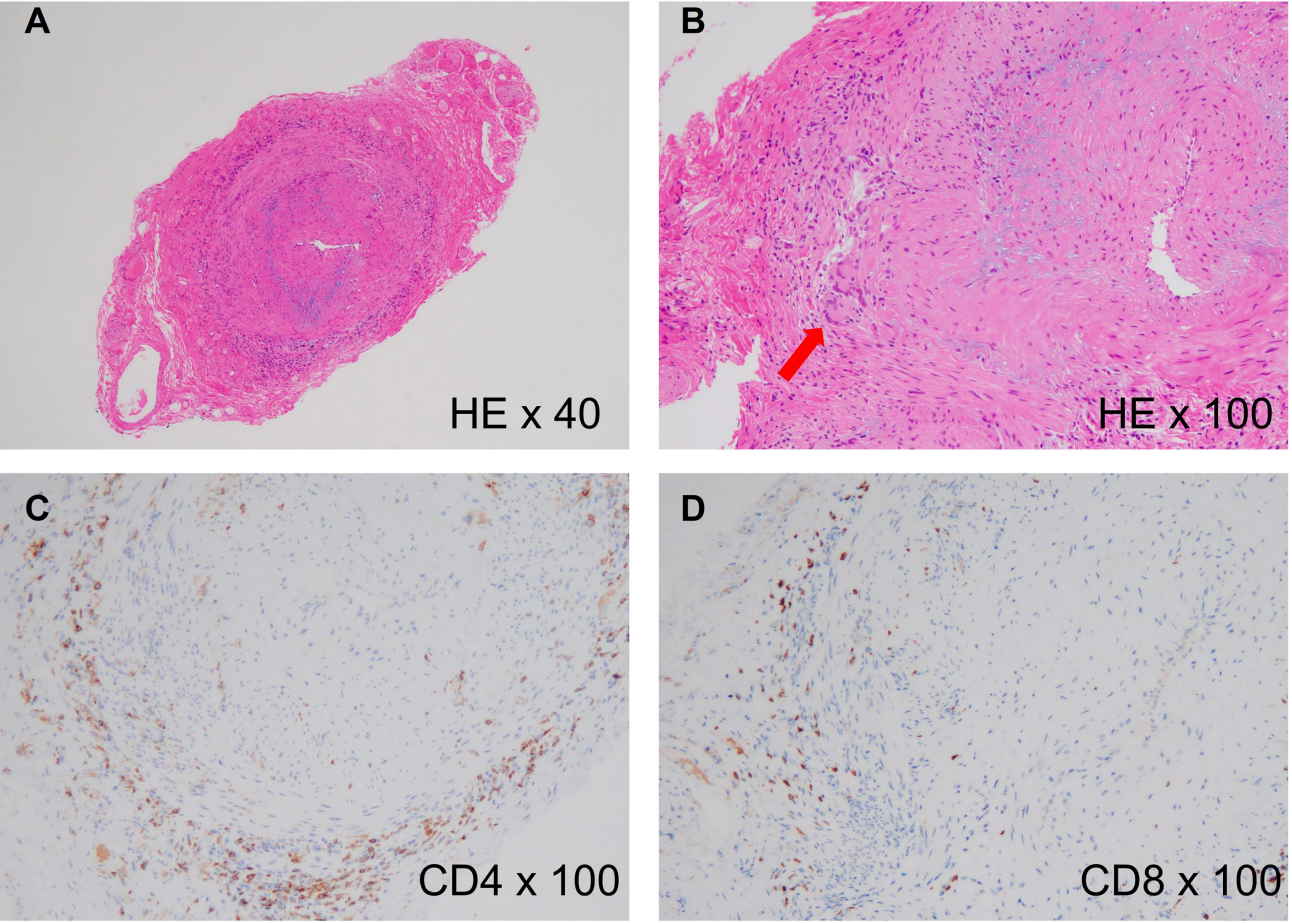
Histology of giant cell arteritis. **(A)** Histological findings of a temporal artery biopsy from an 82-year-old woman with giant cell arteritis (hematoxylin and eosin staining, x40), showing intense cellular infiltration in the adventitia and luminal narrowing due to intimal hyperplasia. **(B)** High-power image of the biopsy (hematoxylin and eosin staining, x100). The red arrow indicates multinucleated giant cells. **(C)** CD4 staining of the biopsy (anti-CD4 staining, x100), showing accumulation of CD4^+^ T cells in the adventitia. **(D)** CD8 staining of the biopsy (Anti-CD8 staining, x100), showing slight accumulation of CD8^+^ T cells in the adventitia.

The pathological findings of these two diseases are indistinguishable and the pathological hallmark of these diseases is chronic granulomatous inflammation, which primarily involves activated CD4^+^ T cells and macrophages (9) (**Figure 1**). Cytokines released from activated CD4^+^ T cells are the main triggers of macrophage activation (10). In addition, genome-wide association studies (GWAS) have revealed that human leukocyte antigens are critically involved in the pathomechanisms of both GCA and TAK (11, 12). These findings suggest that antigen presentation to T cells, particularly CD4^+^ T cells, plays a central role in the development of LVV.

Recent studies have revealed remarkable heterogeneity in CD4^+^ T cells, leading to the discovery of T helper 1 (Th1), Th2, Th9, Th17, Th22, and T follicular helper (Tfh) cells, as well as regulatory T cell subsets (13, 14). Each T cell subset plays a unique role by expressing specific transcription factors and cytokines. Technological advances at the single-cell level have allowed further subdivision of these subsets and have led to the discovery of novel T cell subsets. Accordingly, several T helper cell subsets have been identified in LVV (15, 16). Moreover, aberrant cellular signaling pathways in activated T cells and new T cell subsets, such as tissue-resident memory T cells, have been identified in LVV (17).

In this mini-review, we first outline the current knowledge regarding the immunopathogenesis of LVV, followed by a discussion of the roles of each T cell subset, newly discovered T cell subsets, and aberrant signaling pathways in T cells. Finally, we provide future therapeutic perspectives for LVV based on targeting of T cells.

## The Updated Immunopathogenesis of GCA

Vascular inflammation begins with antigen recognition in vascular dendritic cells (vasDCs) (18, 19). Essentially every artery contains vasDCs, which allow for early detection of foreign antigens. Proliferation of T cells with the shared T cell receptor is confirmed in distinct vascular lesions of GCA (20), which indicates that T cells undergo clonal expansion after recognizing certain antigens. Herpes zoster virus and others have been proposed as antigens (21), but this has not yet been verified. VasDCs express unique Toll-like receptor patterns in each artery (22). In non-inflamed temporal arteritis, the vasDCs are immature and located at the media-adventitia border (23). Once activated, vasDCs expand and express costimulatory molecules such as CD80 and CD86. They also produce excess chemokines and cytokines, which prime naive CD4^+^ T cells and facilitate monocyte migration (24, 25). A recent study demonstrated that defective expression of programmed death ligand 1 in vasDCs also contributes to the maintenance of T cell activation (26, 27). Monocytes then differentiate into tissue macrophages, which are activated by cytokines, particularly interferon (IFN)-γ, released by activated T cells. Activated macrophages in turn start to produce large amounts of cytokines (e.g., IL-6) (28), chemokines (29, 30), proteolytic enzymes (e.g., matrix metalloprotease) (31, 32), and various growth factors, such as vascular endothelial growth factors (VEGF), fibroblast growth factor, and platelet-derived growth factor (33). These growth factors act on endothelial cells (ECs) and vascular smooth muscle cells (VSMCs), transforming them into myofibroblasts, and accelerate intimal hyperplasia and adventitial neoangiogenesis (34–36). Thus, the conventional inflammatory cascade in GCA includes three major players: vasDCs, CD4^+^ T cells, and macrophages. However, several new cell populations have emerged in the pathomechanisms of GCA.

First, neutrophils are not only important mediators of host defenses against pathogens but also contribute to many autoimmune diseases through neutrophil extracellular traps (NETs) (37). Until recently, the role of neutrophils has been underestimated because of the rarity of vascular lesions in GCA; however, mapping of immune cell populations from GCA patients has shown that immature neutrophils generate high levels of reactive oxygen species and enhance protein oxidation, leading to endothelial barrier dysfunction in vascular lesions (38). These findings link the function of immature neutrophils to disease mechanisms.

Second, intimal hyperplasia is thought to be caused by transformation and expansion of ECs and VSMCs into myofibroblasts. Recently, however, it has been proposed that fibroblasts located in the adventitia start to produce α smooth muscle actin and collagen by an unknown trigger, phenotypically change into myofibroblasts, and migrate toward the intimal layer (39). As fibroblasts are also abundant in the vascular lesions of TAK (40), these cells could be therapeutically targeted in LVV.

## Vasculitogenic T Cells in GCA

### Th1 Cells

**Figure 2:**
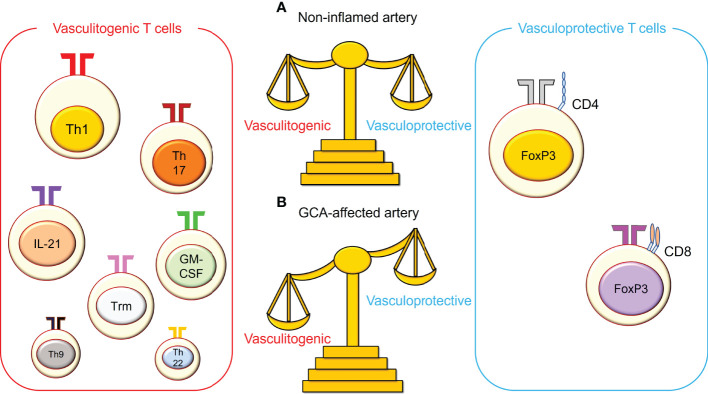
The imbalance between vasculitogenic T cells and vasculoprotective T cells in giant cell arteritis. **(A)** In non-inflamed arteries, vasculitogenic T cells and vasculoprotective T cells are well balanced in the blood, and both cell types are rare in the vasculature. **(B)** In giant cell arteritis (GCA), this balance is perturbed, and vasculitogenic T cells accumulate, while the number of vasculoprotective T cells decreases in the tissue and the blood. Vasculitogenic T cells include T helper 1 (Th1) cells, Th17 cells, IL-21-producing T cells, granulocyte macrophage-colony stimulating factor (GM-CSF)-producing T cells, tissue-resident memory T (Trm) cells, Th9 cells, and Th22 cells. On the other hand, vasculoprotective T cells include CD4^+^ regulatory T cells and CD8^+^ regulatory T cells.

Analysis of the T cell population in vascular tissues and the circulatory system suggests polyclonal T cell activation (41–43)(**Figure 2**). Among these, Th1 cells appear to be the dominant cell population and are highly enriched in vascular tissues and the circulatory system in GCA (15, 44). Naive CD4^+^ T cells are induced to express transcription factor T-bet and differentiate into Th1 cells in the presence of IL-12 (13), which is abundant in GCA-affected arteries (45). IFN-γ released by Th1 cells not only stimulates macrophages and provides protective immunity against intracellular pathogens but also affects ECs, VSMCs, and fibroblasts. Although IFN-γ impairs the proliferation and survival of ECs in the tumor microenvironment (46, 47), it promotes angiogenesis *via* VEGF produced by tissue macrophages in GCA (10). IFN-γ induces VSCM proliferation in atherosclerosis (48). The direct effect on fibroblasts residing in blood vessels is unknown. However, upon stimulation with IFN-γ, synovial fibroblasts upregulate MHC class II expression and increase IL-6 production (49). Thus, IFN-γ derived from Th1 cells is implicated in several pathogenic events in GCA.

### Th17 Cells

Compared to Th1 cells, all other functional T cell lineages occur at much lower frequencies (42), although Th17 cell numbers are increased in GCA (15). Naive CD4^+^ T cells express the master transcription factor RORγt and differentiate into Th17 cells in the presence of IL-6 and transforming growth factor β, which are abundant in the vascular lesions (50, 51). IL-23 appears to function in the expansion and maturation of Th17 cells at a late stage (13). Th17 cells produce IL-17, IL-21, and IL-22, and the IL-17 family includes six isoforms (IL-17A to IL-17F) (52). IL-17A not only provides host defense against extracellular pathogens, including bacteria, fungi, and mycobacteria but also participates in autoimmunity (53, 54). IL-17A acts on ECs, resulting in the secretion of proinflammatory cytokines, such as IL-6 and chemokines (55). IL-17 is also involved in the proinflammatory response of VSMCs, inducing the release of cytokines such as IL-6 and granulocyte–macrophage colony-stimulating factor (GM-CSF) (56).

What is the molecular basis for the increase in Th1 and Th17 cells in GCA? A recent study revealed that the VEGF-NOTCH1 axis plays a role in skewed T cell polarization (57). VEGF is primarily derived from tissue macrophages and is enriched in GCA plasma (58). The innermost ECs of the vasa vasorum respond to VEGF and upregulate the expression of NOTCH1 ligand, which in turn stimulates NOTCH1 receptor expressed on GCA CD4^+^ T cells. This NOTCH1 ligand-NOTCH1 interaction induces activation of the mechanistic target of rapamycin (mTOR), shifting T cell differentiation toward Th1 and Th17 cells (57). mTOR is a serine/threonine protein kinase that constitutes the catalytic subunit of two distinct complexes: mTOR complex 1 (mTORC1) and mTORC2 (59). The mTOR pathway integrates a diverse set of environmental factors to direct cellular growth and is implicated in metabolic disorders, neurodegeneration, cancer, and aging (60). Recent studies have shown that mTORC1 regulates the differentiation of T helper cells and is involved in Th1 and Th17 development (61). Thus, the VEGF-NOTCH1-mTORC1 axis contributes to skewed T cell differentiation.

### IL-21-Producing CD4^+^ T Cells

The number of IL-21-producing CD4^+^ T cells is also increased in vascular lesions and in the blood (44). IL-21-producing CD4^+^ T cells account for approximately 2.5% of the peripheral blood cells in healthy individuals, whereas this proportion increases to approximately 8% in patients with GCA, which cannot be explained by the frequency of Th17 cells. IL-21 is the main cytokine produced by Tfh cells, which help B cells to secrete IgG antibodies, but the Tfh-B cell signature is not upregulated in GCA (62). Thus, the cellular origin of IL-21 remains unclear. However, since IL-21 is able to shift T cell differentiation toward Th1 and Th17 phenotypes and decrease the number of regulatory T cells (Tregs) (44), this cytokine could be a therapeutic target for GCA.

### GM-CSF-Producing T Cells and Other T Helper Cells

Recently, GM-CSF has emerged as a key cytokine in the pathogenesis of GCA (63–65). GM-CSF and GM-CSF receptors are highly expressed in GCA-affected arteries. Although Th1 and Th17 cells are the major sources of GM-CSF in the joints with rheumatoid arthritis (RA) (66, 67), GM-CSF is produced by endothelial cells, macrophages, and T cells in vascular lesions of GCA. In ex vivo cultured arteries, anti-GM-CSF receptor antibodies have shown promise, decreasing T cell and macrophage numbers as well as proinflammatory cytokine expression (63). Other T helper cell subsets, such as Th9 and Th22 cells, are also implicated in the amplification of vascular inflammation (68, 69), although their precise role is still unclear.

### Tissue-Resident Memory T Cells

The results of a study that enrolled patients with GCA confirmed by temporal artery biopsy (TAB) and prospectively performed a second TAB from the contralateral side to the first TAB showed residual inflammation in approximately half of the patients even after one year of treatment (70). The pathological analysis showing that T cells were the main residual cells prompted us to investigate tissue-resident memory T (Trm) cells. A subset of effector T cells resides in lymphoid and non-lymphoid tissues without recirculation through the blood and gives rise to Trm cells (71, 72). A key feature of Trm cells is their ability to be retained in barrier tissues for prolonged periods of time and their rapid response when encountering the same antigen (73). Trm cells are characterized by the expression of C-type lectin CD69 and integrin CD103 (74). In our mouse model of LVV, approximately 10% of the CD4^+^ T cells infiltrating the vascular tissue expressed CD103. Interestingly, tissue residency of Trm cells requires signals from the JAK-STAT pathway and CD28 stimulation from tissue niches (17, 75). Further characterization of these Trm cells may lead to the development of therapeutic strategies to specifically eliminate them.

## Vasculoprotective T Cells in GCA

Naturally occurring CD4^+^ Tregs, which express the transcription factor FoxP3 in the nucleus and CD25 on the cell surface, are a functionally distinct T cell subset actively engaged in the maintenance of immunological tolerance (76). Since IL-6 and IL-21 have been reported to inhibit Treg differentiation (44, 77) and these cytokines are highly enriched in the plasma of patients with GCA, the number of CD4^+^ Tregs is reduced in patients with GCA compared to healthy controls (44). However, accumulating evidence suggests that tocilizumab (TCZ), an IL-6 receptor inhibitor, restores not only the number of CD4^+^ Tregs but also the function of these cells (78–80). Accordingly, TCZ decreases relapse and has a steroid-tapering effect on GCA (81).

While CD4^+^ Tregs are well recognized and established, their CD8^+^ counterparts are still controversial in many regards, including their phenotypic identity and mechanisms of suppression (82); however, the immunosuppressive effects of CD8^+^ Tregs have been proven in some experimental models such as inflammatory bowel disease and graft-versus-host disease (83, 84). Compared with younger individuals, the number of CD8^+^ Tregs is reduced in the elderly, and these cells are significantly reduced in number and function in GCA patients (85). The functional defect of CD8^+^ Tregs is attributed to inadequate release of exosomes containing NADPH oxidase 2 (NOX2), which inhibits neighboring CD4^+^ T cell activation by blocking the phosphorylation of ZAP-70, a proximal molecule directly involved in T cell receptor signaling (85, 86).

## Vasculitogenic and Vasculoprotective T Cells in TAK

Similar to GCA, an increase in Th1 and Th17 cells has been reported in TAK (16, 87). This increase may stem from overactivation of the NOTCH1-mTOR pathway (88, 89) and/or an increase in the number of CD4^+^ IL-21-producing T cells (89, 90). In parallel, a decrease in CD4^+^ Treg numbers has also been documented (91). In contrast, unlike GCA, microarray analysis has demonstrated that the Tfh signature that includes CXCR5 and CCR6 is significantly increased in the blood and the aorta (62). Tfh cells may be partly responsible for the elevated levels of IL-21 in TAK. Moreover, it has been reported that CD8^+^ T cell infiltration is more common in TAK than in GCA (92). Recent immunophenotyping analysis using flow cytometry has added another piece of evidence showing that circulating CD8^+^ T cells were increased only during the active phase in TAK, but the number of these cells in GCA was stable irrespective of the disease activity (93). The contribution of CD8^+^ T cells to the pathomechanisms of TAK may be greater than their contribution in GCA.

## Discussion

CD4^+^ T cells undoubtedly play a central role in LVV pathogenesis. Additionally, CD8^+^ T cells and natural killer cells actively engage in the disease mechanism of TAK, making it more complex than that of GCA (40). Indeed, abatacept (ABT), which selectively inhibits T cell activation by blocking the co-stimulatory signal, has been shown to improve the disease activity of RA and reduce the relapse rate in patients with GCA (94), but it failed to exhibit efficacy in TAK (95). However, the high relapse rate even in the ABT treatment arm in patients with GCA prompts us to explore better therapeutic options for LVV.

Considering the disease mechanism, the VEGF-NOTCH1-mTOR pathway as well as T cell polarizing cytokines, such as IL-12, IL-23, and IL-21, could be therapeutic targets to correct biased CD4^+^ T cell differentiation and suppress LVV. Anti-VEGF antibody, an mTOR inhibitor, and an IL-12/IL-23 inhibitor (e.g., ustekinumab) are on the market. Ustekinumab has been tested for GCA and TAK (96, 97), but the results obtained to date are not encouraging (98, 99).

Other therapeutic options include inhibition of released cytokines, including IL-17, GM-CSF, and IFN-γ. The efficacy and safety of anti-IL-17 and anti-GM-CSF receptor antibodies against GCA are being actively pursued in clinical trials, and the results obtained to date appear promising (100, 101). Signaling downstream of GM-CSF and IFN-γ involves the JAK-STAT pathway, and the efficacy of JAK inhibitors is widely recognized in RA (102), which raises expectations for the treatment of LVV (103). Indeed, in addition to IFN-γ, type I IFN is also highly expressed in the vascular lesions, and there have been several reports of increased activation of the JAK-STAT pathway in GCA- and TAK-T cells (17, 104–106).

Furthermore, GWAS has identified *IL-12B* as a susceptibility gene for TAK (12, 107), and the *IL-12B* risk allele is associated with vascular damage in TAK (108). IL-12 relies on the JAK-STAT pathway for intracellular signal transduction. Although the results of ustekinumab are not encouraging, JAK inhibitors may have potential for treating TAK (109).

In conclusion, recent research advances have shed new light on the role of T cells in the disease mechanisms of LVV. Several treatment options targeting T cells are expected to emerge in the near future.

## Author Contributions

RW drafted the manuscript. MH revised and finalized the manuscript. All authors contributed to the article and approved the submitted version.

## Funding

This work was in part supported by JSPS KAKENHI Grant Number 20K17418 and a grant-in-aid of the Cardiovascular Research Fund, Tokyo, Japan and the Japan Rheumatism Foundation, Tokyo, Japan to RW.

## Conflict of Interest

The authors declare that the research was conducted in the absence of any commercial or financial relationships that could be construed as a potential conflict of interest.

## Publisher’s Note

All claims expressed in this article are solely those of the authors and do not necessarily represent those of their affiliated organizations, or those of the publisher, the editors and the reviewers. Any product that may be evaluated in this article, or claim that may be made by its manufacturer, is not guaranteed or endorsed by the publisher.

## References

[1] Jennette JC Falk RJ Bacon PA Basu N Cid MC Ferrario F. 2012 Revised International Chapel Hill Consensus Conference Nomenclature of Vasculitides. Arthritis Rheum (2013) 65:1–11. doi: 10.1002/art.37715 23045170

[2] Gloor AD Berry GJ Goronzy JJ Weyand CM. Age as a Risk Factor in Vasculitis. Semin Immunopathol (2022) 44(3):281–301. doi: 10.1007/s00281-022-00911-1 35141865 PMC9064861

[3] Van Der Geest KSM Sandovici M Brouwer E Mackie SL. Diagnostic Accuracy of Symptoms, Physical Signs, and Laboratory Tests for Giant Cell Arteritis: A Systematic Review and Meta-Analysis. JAMA Intern Med (2020) 180:1295–304. doi: 10.1001/jamainternmed.2020.3050 PMC7432275 32804186

[4] Okazaki S Watanabe R Kondo H Kudo M Harigae H Fujii H. High Relapse Rate in Patients With Polymyalgia Rheumatica Despite the Combination of Immunosuppressants and Prednisolone: A Single Center Experience of 89 Patients. Tohoku J Exp Med (2020) 251:125–33. doi: 10.1620/tjem.251.125 32581186

[5] Numano F. Differences in Clinical Presentation and Outcome in Different Countries for Takayasu's Arteritis. Curr Opin Rheumatol (1997) 9:12–5. doi: 10.1097/00002281-199701000-00003 9110128

[6] Yoshida M Watanabe R Ishii T Machiyama T Akita K Fujita Y. Retrospective Analysis of 95 Patients With Large Vessel Vasculitis: A Single Center Experience. Int J Rheum Dis (2016) 19:87–94. doi: 10.1111/1756-185X.12777 26443306

[7] Elbendary A Abdel-Halim MRE Ragab G. Updates in Cutaneous Manifestations of Systemic Vasculitis. Curr Opin Rheumatol (2022) 34:25–32. doi: 10.1097/BOR.0000000000000847 34690279

[8] Watanabe R Ishii T Nakamura K Shirai T Fujii H Saito S. Ulcerative Colitis Is Not a Rare Complication of Takayasu Arteritis. Mod Rheumatol (2014) 24:372–3. doi: 10.3109/14397595.2013.854045 24593217

[9] Weyand CM Goronzy JJ. Immune Mechanisms in Medium and Large-Vessel Vasculitis. Nat Rev Rheumatol (2013) 9:731–40. doi: 10.1038/nrrheum.2013.161 PMC4277683 24189842

[10] Kaiser M Younge B Bjornsson J Goronzy JJ Weyand CM. Formation of New Vasa Vasorum in Vasculitis. Production of Angiogenic Cytokines by Multinucleated Giant Cells. Am J Pathol (1999) 155:765–74. doi: 10.1016/S0002-9440(10)65175-9 PMC1866901 10487834

[11] Carmona FD Vaglio A Mackie SL Hernandez-Rodriguez J Monach PA Castaneda S. A Genome-Wide Association Study Identifies Risk Alleles in Plasminogen and P4HA2 Associated With Giant Cell Arteritis. Am J Hum Genet (2017) 100:64–74. doi: 10.1016/j.ajhg.2016.11.013 28041642 PMC5223025

[12] Terao C Yoshifuji H Matsumura T Naruse TK Ishii T Nakaoka Y. Genetic Determinants and an Epistasis of LILRA3 and HLA-B*52 in Takayasu Arteritis. Proc Natl Acad Sci USA (2018) 115:13045–50. doi: 10.1073/pnas.1808850115 PMC6304955 30498034

[13] Dong C. Cytokine Regulation and Function in T Cells. Annu Rev Immunol (2021) 39:51–76. doi: 10.1146/annurev-immunol-061020-053702 33428453

[14] Sakaguchi S Vignali DA Rudensky AY Niec RE Waldmann H. The Plasticity and Stability of Regulatory T Cells. Nat Rev Immunol (2013) 13:461–7. doi: 10.1038/nri3464 23681097

[15] Deng J Younge BR Olshen RA Goronzy JJ Weyand CM. Th17 and Th1 T-Cell Responses in Giant Cell Arteritis. Circulation (2010) 121:906–15. doi: 10.1161/CIRCULATIONAHA.109.872903 PMC2837465 20142449

[16] Saadoun D Garrido M Comarmond C Desbois AC Domont F Savey L. Th1 and Th17 Cytokines Drive Inflammation in Takayasu Arteritis. Arthritis Rheumatol (2015) 67:1353–60. doi: 10.1002/art.39037 25604824

[17] Zhang H Watanabe R Berry GJ Tian L Goronzy JJ Weyand CM. Inhibition of JAK-STAT Signaling Suppresses Pathogenic Immune Responses in Medium and Large Vessel Vasculitis. Circulation (2018) 137:1934–48. doi: 10.1161/CIRCULATIONAHA.117.030423 PMC5930040 29254929

[18] Samson M Corbera-Bellalta M Audia S Planas-Rigol E Martin L Cid MC. Recent Advances in Our Understanding of Giant Cell Arteritis Pathogenesis. Autoimmun Rev (2017) 16:833–44. doi: 10.1016/j.autrev.2017.05.014 28564617

[19] Watanabe R Goronzy JJ Berry G Liao YJ Weyand CM. Giant Cell Arteritis: From Pathogenesis to Therapeutic Management. Curr Treatm Opt Rheumatol (2016) 2:126–37. doi: 10.1007/s40674-016-0043-x PMC4902281 27298757

[20] Martinez-Taboada V Hunder NN Hunder GG Weyand CM Goronzy JJ. Recognition of Tissue Residing Antigen by T Cells in Vasculitic Lesions of Giant Cell Arteritis. J Mol Med (Berl) (1996) 74:695–703. doi: 10.1007/s001090050074 8956156

[21] Gilden D White T Khmeleva N Heintzman A Choe A Boyer PJ. Prevalence and Distribution of VZV in Temporal Arteries of Patients With Giant Cell Arteritis. Neurology (2015) 84:1948–55. doi: 10.1212/WNL.0000000000001409 PMC4433460 25695965

[22] Pryshchep O Ma-Krupa W Younge BR Goronzy JJ Weyand CM. Vessel-Specific Toll-Like Receptor Profiles in Human Medium and Large Arteries. Circulation (2008) 118:1276–84. doi: 10.1161/CIRCULATIONAHA.108.789172 PMC2748975 18765390

[23] Ma-Krupa W Jeon MS Spoerl S Tedder TF Goronzy JJ Weyand CM. Activation of Arterial Wall Dendritic Cells and Breakdown of Self-Tolerance in Giant Cell Arteritis. J Exp Med (2004) 199:173–83. doi: 10.1084/jem.20030850 PMC2211768 14734523

[24] Krupa WM Dewan M Jeon MS Kurtin PJ Younge BR Goronzy JJ. Trapping of Misdirected Dendritic Cells in the Granulomatous Lesions of Giant Cell Arteritis. Am J Pathol (2002) 161:1815–23. doi: 10.1016/S0002-9440(10)64458-6 PMC1850804 12414528

[25] Wagner AD Wittkop U Prahst A Schmidt WA Gromnica-Ihle E Vorpahl K. Dendritic Cells Co-Localize With Activated CD4+ T Cells in Giant Cell Arteritis. Clin Exp Rheumatol (2003) 21:185–92. 12747272

[26] Watanabe R Zhang H Berry G Goronzy JJ Weyand CM. Immune Checkpoint Dysfunction in Large and Medium Vessel Vasculitis. Am J Physiol Heart Circ Physiol (2017) 312:H1052–h1059. doi: 10.1152/ajpheart.00024.2017 28314758 PMC5451585

[27] Zhang H Watanabe R Berry GJ Vaglio A Liao YJ Warrington KJ. Immunoinhibitory Checkpoint Deficiency in Medium and Large Vessel Vasculitis. Proc Natl Acad Sci U S A (2017) 114:E970–e979. doi: 10.1073/pnas.1616848114 28115719 PMC5307483

[28] Wagner AD Goronzy JJ Weyand CM. Functional Profile of Tissue-Infiltrating and Circulating CD68+ Cells in Giant Cell Arteritis. Evidence for Two Components of the Disease. J Clin Invest (1994) 94:1134–40. doi: 10.1172/JCI117428 PMC295180 8083354

[29] Corbera-Bellalta M Planas-Rigol E Lozano E Terrades-Garcia N Alba MA Prieto-Gonzalez S. Blocking Interferon Gamma Reduces Expression of Chemokines CXCL9, CXCL10 and CXCL11 and Decreases Macrophage Infiltration in Ex Vivo Cultured Arteries From Patients With Giant Cell Arteritis. Ann Rheum Dis (2016) 75:1177–86. doi: 10.1136/annrheumdis-2015-208371 26698852

[30] Watanabe R Hilhorst M Zhang H Zeisbrich M Berry GJ Wallis BB. Glucose Metabolism Controls Disease-Specific Signatures of Macrophage Effector Functions. JCI Insight (2018) 3(20):e123047. doi: 10.1172/jci.insight.123047 PMC6237479 30333306

[31] Van Sleen Y Jiemy WF Pringle S Van Der Geest KSM Abdulahad WH Sandovici M. A Distinct Macrophage Subset Mediating Tissue Destruction and Neovascularization in Giant Cell Arteritis: Implication of the YKL-40/Interleukin-13 Receptor Alpha2 Axis. Arthritis Rheumatol (2021) 73:2327–37. doi: 10.1002/art.41887 PMC9298326 34105308

[32] Watanabe R Maeda T Zhang H Berry GJ Zeisbrich M Brockett R. MMP (Matrix Metalloprotease)-9-Producing Monocytes Enable T Cells to Invade the Vessel Wall and Cause Vasculitis. Circ Res (2018) 123:700–15. doi: 10.1161/CIRCRESAHA.118.313206 PMC6202245 29970365

[33] Weyand CM Watanabe R Zhang H Akiyama M Berry GJ Goronzy JJ. Cytokines, Growth Factors and Proteases in Medium and Large Vessel Vasculitis. Clin Immunol (2019) 206:33–41. doi: 10.1016/j.clim.2019.02.007 30772599 PMC6693995

[34] Kaiser M Weyand CM Bjornsson J Goronzy JJ. Platelet-Derived Growth Factor, Intimal Hyperplasia, and Ischemic Complications in Giant Cell Arteritis. Arthritis Rheum (1998) 41:623–33. doi: 10.1002/1529-0131(199804)41:4<623::AID-ART9>3.0.CO;2-6 9550471

[35] Lozano E Segarra M Garcia-Martinez A Hernandez-Rodriguez J Cid MC. Imatinib Mesylate Inhibits *In Vitro* and Ex Vivo Biological Responses Related to Vascular Occlusion in Giant Cell Arteritis. Ann Rheum Dis (2008) 67:1581–8. doi: 10.1136/ard.2007.070805 17584806

[36] Pulsatelli L Boiardi L Assirelli E Pazzola G Muratore F Addimanda O. Imbalance Between Angiogenic and Anti-Angiogenic Factors in Sera From Patients With Large-Vessel Vasculitis. Clin Exp Rheumatol (2020) 38 Suppl 124:23–30. 31573481

[37] Michailidou D Mustelin T Lood C. Role of Neutrophils in Systemic Vasculitides. Front Immunol (2020) 11:619705. doi: 10.3389/fimmu.2020.619705 33391289 PMC7774018

[38] Wang L Ai Z Khoyratty T Zec K Eames HL Van Grinsven E. ROS-Producing Immature Neutrophils in Giant Cell Arteritis are Linked to Vascular Pathologies. JCI Insight (2020) 5(20):e139163. doi: 10.1172/jci.insight.139163 PMC7605529 32960815

[39] Parreau S Vedrenne N Regent A Richard L Sindou P Mouthon L. An Immunohistochemical Analysis of Fibroblasts in Giant Cell Arteritis. Ann Diagn Pathol (2021) 52:151728. doi: 10.1016/j.anndiagpath.2021.151728 33798926

[40] Watanabe R Berry GJ Liang DH Goronzy JJ Weyand CM. Pathogenesis of Giant Cell Arteritis and Takayasu Arteritis-Similarities and Differences. Curr Rheumatol Rep (2020) 22:68. doi: 10.1007/s11926-020-00948-x 32845392 PMC9112376

[41] Schaufelberger C Stemme S Andersson R Hansson GK. T Lymphocytes in Giant Cell Arteritic Lesions are Polyclonal Cells Expressing Alpha Beta Type Antigen Receptors and VLA-1 Integrin Receptors. Clin Exp Immunol (1993) 91:421–8. doi: 10.1111/j.1365-2249.1993.tb05919.x PMC1554726 8383021

[42] Watanabe R Hosgur E Zhang H Wen Z Berry G Goronzy JJ. Pro-Inflammatory and Anti-Inflammatory T Cells in Giant Cell Arteritis. Joint Bone Spine (2017) 84:421–6. doi: 10.1016/j.jbspin.2016.07.005 PMC5639893 27663755

[43] Weyand CM Schonberger J Oppitz U Hunder NN Hicok KC Goronzy JJ. Distinct Vascular Lesions in Giant Cell Arteritis Share Identical T Cell Clonotypes. J Exp Med (1994) 179:951–60. doi: 10.1084/jem.179.3.951 PMC2191412 8113687

[44] Terrier B Geri G Chaara W Allenbach Y Rosenzwajg M Costedoat-Chalumeau N. Interleukin-21 Modulates Th1 and Th17 Responses in Giant Cell Arteritis. Arthritis Rheum (2012) 64:2001–11. doi: 10.1002/art.34327 22147555

[45] Espigol-Frigole G Planas-Rigol E Lozano E Corbera-Bellalta M Terrades-Garcia N Prieto-Gonzalez S. Expression and Function of IL12/23 Related Cytokine Subunits (P35, P40, and P19) in Giant-Cell Arteritis Lesions: Contribution of P40 to Th1- and Th17-Mediated Inflammatory Pathways. Front Immunol (2018) 9:809. doi: 10.3389/fimmu.2018.00809 29731755 PMC5920281

[46] Beatty G Paterson Y. IFN-Gamma-Dependent Inhibition of Tumor Angiogenesis by Tumor-Infiltrating CD4+ T Cells Requires Tumor Responsiveness to IFN-Gamma. J Immunol (2001) 166:2276–82. doi: 10.4049/jimmunol.166.4.2276 11160282

[47] Kammertoens T Friese C Arina A Idel C Briesemeister D Rothe M. Tumour Ischaemia by Interferon-Gamma Resembles Physiological Blood Vessel Regression. Nature (2017) 545:98–102. doi: 10.1038/nature22311 28445461 PMC5567674

[48] Wang Y Bai Y Qin L Zhang P Yi T Teesdale SA. Interferon-Gamma Induces Human Vascular Smooth Muscle Cell Proliferation and Intimal Expansion by Phosphatidylinositol 3-Kinase Dependent Mammalian Target of Rapamycin Raptor Complex 1 Activation. Circ Res (2007) 101:560–9. doi: 10.1161/CIRCRESAHA.107.151068 17656678

[49] Kato M. New Insights Into IFN-Gamma in Rheumatoid Arthritis: Role in the Era of JAK Inhibitors. Immunol Med (2020) 43:72–8. doi: 10.1080/25785826.2020.1751908 32338187

[50] Roche NE Fulbright JW Wagner AD Hunder GG Goronzy JJ Weyand CM. Correlation of Interleukin-6 Production and Disease Activity in Polymyalgia Rheumatica and Giant Cell Arteritis. Arthritis Rheum (1993) 36:1286–94. doi: 10.1002/art.1780360913 8216422

[51] Weyand CM Hicok KC Hunder GG Goronzy JJ. Tissue Cytokine Patterns in Patients With Polymyalgia Rheumatica and Giant Cell Arteritis. Ann Intern Med (1994) 121:484–91. doi: 10.7326/0003-4819-121-7-199410010-00003 8067646

[52] Miossec P Kolls JK. Targeting IL-17 and TH17 Cells in Chronic Inflammation. Nat Rev Drug Discovery (2012) 11:763–76. doi: 10.1038/nrd3794 23023676

[53] Hirota K Hashimoto M Yoshitomi H Tanaka S Nomura T Yamaguchi T. T Cell Self-Reactivity Forms a Cytokine Milieu for Spontaneous Development of IL-17+ Th Cells That Cause Autoimmune Arthritis. J Exp Med (2007) 204:41–7. doi: 10.1084/jem.20062259 PMC2118414 17227914

[54] Miossec P Korn T Kuchroo VK. Interleukin-17 and Type 17 Helper T Cells. N Engl J Med (2009) 361:888–98. doi: 10.1056/NEJMra0707449 19710487

[55] Fossiez F Djossou O Chomarat P Flores-Romo L Ait-Yahia S Maat C. T Cell Interleukin-17 Induces Stromal Cells to Produce Proinflammatory and Hematopoietic Cytokines. J Exp Med (1996) 183:2593–603. doi: 10.1084/jem.183.6.2593 PMC2192621 8676080

[56] Pietrowski E Bender B Huppert J White R Luhmann HJ Kuhlmann CR. Pro-Inflammatory Effects of Interleukin-17A on Vascular Smooth Muscle Cells Involve NAD(P)H- Oxidase Derived Reactive Oxygen Species. J Vasc Res (2011) 48:52–8. doi: 10.1159/000317400 20606471

[57] Wen Z Shen Y Berry G Shahram F Li Y Watanabe R. The Microvascular Niche Instructs T Cells in Large Vessel Vasculitis *via* the VEGF-Jagged1-Notch Pathway. Sci Transl Med (2017) 9(399):eaal3322. doi: 10.1126/scitranslmed.aal3322 PMC5708299 28724574

[58] Baldini M Maugeri N Ramirez GA Giacomassi C Castiglioni A Prieto-Gonzalez S. Selective Up-Regulation of the Soluble Pattern-Recognition Receptor Pentraxin 3 and of Vascular Endothelial Growth Factor in Giant Cell Arteritis: Relevance for Recent Optic Nerve Ischemia. Arthritis Rheum (2012) 64:854–65. doi: 10.1002/art.33411 21989653

[59] Liu GY Sabatini DM. mTOR at the Nexus of Nutrition, Growth, Ageing and Disease. Nat Rev Mol Cell Biol (2020) 21:183–203. doi: 10.1038/s41580-019-0199-y 31937935 PMC7102936

[60] Zoncu R Efeyan A Sabatini DM. mTOR: From Growth Signal Integration to Cancer, Diabetes and Ageing. Nat Rev Mol Cell Biol (2011) 12:21–35. doi: 10.1038/nrm3025 21157483 PMC3390257

[61] Delgoffe GM Pollizzi KN Waickman AT Heikamp E Meyers DJ Horton MR. The Kinase mTOR Regulates the Differentiation of Helper T Cells Through the Selective Activation of Signaling by Mtorc1 and Mtorc2. Nat Immunol (2011) 12:295–303. doi: 10.1038/ni.2005 21358638 PMC3077821

[62] Desbois AC Regnier P Quiniou V Lejoncour A Maciejewski-Duval A Comarmond C. Specific Follicular Helper T Cell Signature in Takayasu Arteritis. Arthritis Rheumatol (2021) 73:1233–43. doi: 10.1002/art.41672 33538119

[63] Corbera-Bellalta M Alba-Rovira R Muralidharan S Espigol-Frigole G Rios-Garces R Marco-Hernandez J. Blocking GM-CSF Receptor Alpha With Mavrilimumab Reduces Infiltrating Cells, Pro-Inflammatory Markers and Neoangiogenesis in Ex Vivo Cultured Arteries From Patients With Giant Cell Arteritis. Ann Rheum Dis (2022) 81:524–36. doi: 10.1136/annrheumdis-2021-220873 PMC8921590 35045965

[64] Jiemy WF Van Sleen Y Van Der Geest KS Ten Berge HA Abdulahad WH Sandovici M. Distinct Macrophage Phenotypes Skewed by Local Granulocyte Macrophage Colony-Stimulating Factor (GM-CSF) and Macrophage Colony-Stimulating Factor (M-CSF) are Associated With Tissue Destruction and Intimal Hyperplasia in Giant Cell Arteritis. Clin Transl Immunol (2020) 9:e1164. doi: 10.1002/cti2.1164 PMC7453134 32884747

[65] Wagner AD Wittkop U Thalmann J Willmen T Godecke V Hodam J. Glucocorticoid Effects on Tissue Residing Immune Cells in Giant Cell Arteritis: Importance of GM-CSF. Front Med (Lausanne) (2021) 8:709404. doi: 10.3389/fmed.2021.709404 34557501 PMC8452956

[66] Hirota K Hashimoto M Ito Y Matsuura M Ito H Tanaka M. Autoimmune Th17 Cells Induced Synovial Stromal and Innate Lymphoid Cell Secretion of the Cytokine GM-CSF to Initiate and Augment Autoimmune Arthritis. Immunity (2018) 48:1220–1232.e5. doi: 10.1016/j.immuni.2018.04.009 29802020 PMC6024031

[67] Yamada H Haraguchi A Sakuraba K Okazaki K Fukushi JI Mizu-Uchi H. Th1 Is the Predominant Helper T Cell Subset That Produces GM-CSF in the Joint of Rheumatoid Arthritis. RMD Open (2017) 3:e000487. doi: 10.1136/rmdopen-2017-000487 28955490 PMC5604604

[68] Ciccia F Rizzo A Guggino G Cavazza A Alessandro R Maugeri R. Difference in the Expression of IL-9 and IL-17 Correlates With Different Histological Pattern of Vascular Wall Injury in Giant Cell Arteritis. Rheumatol (Oxford) (2015) 54:1596–604. doi: 10.1093/rheumatology/kev102 25862016

[69] Zerbini A Muratore F Boiardi L Ciccia F Bonacini M Belloni L. Increased Expression of Interleukin-22 in Patients With Giant Cell Arteritis. Rheumatol (Oxford) (2018) 57:64–72. doi: 10.1093/rheumatology/kex334 28968695

[70] Maleszewski JJ Younge BR Fritzlen JT Hunder GG Goronzy JJ Warrington KJ. Clinical and Pathological Evolution of Giant Cell Arteritis: A Prospective Study of Follow-Up Temporal Artery Biopsies in 40 Treated Patients. Mod Pathol (2017) 30:788–96. doi: 10.1038/modpathol.2017.10 PMC5650068 28256573

[71] Iijima N Iwasaki A. Tissue Instruction for Migration and Retention of TRM Cells. Trends Immunol (2015) 36:556–64. doi: 10.1016/j.it.2015.07.002 PMC4567393 26282885

[72] Masopust D Soerens AG. Tissue-Resident T Cells and Other Resident Leukocytes. Annu Rev Immunol (2019) 37:521–46. doi: 10.1146/annurev-immunol-042617-053214 PMC7175802 30726153

[73] Takamura S. Niches for the Long-Term Maintenance of Tissue-Resident Memory T Cells. Front Immunol (2018) 9:1214. doi: 10.3389/fimmu.2018.01214 29904388 PMC5990602

[74] Hirahara K Kokubo K Aoki A Kiuchi M Nakayama T. The Role of CD4(+) Resident Memory T Cells in Local Immunity in the Mucosal Tissue - Protection Versus Pathology. Front Immunol (2021) 12:616309. doi: 10.3389/fimmu.2021.616309 33968018 PMC8097179

[75] Zhang H Watanabe R Berry GJ Nadler SG Goronzy JJ Weyand CM. CD28 Signaling Controls Metabolic Fitness of Pathogenic T Cells in Medium and Large Vessel Vasculitis. J Am Coll Cardiol (2019) 73:1811–23. doi: 10.1016/j.jacc.2019.01.049 PMC6709860 30975299

[76] Sakaguchi S Mikami N Wing JB Tanaka A Ichiyama K Ohkura N. Regulatory T Cells and Human Disease. Annu Rev Immunol (2020) 38:541–66. doi: 10.1146/annurev-immunol-042718-041717 32017635

[77] Kishimoto T Kang S. IL-6 Revisited: From Rheumatoid Arthritis to CAR T Cell Therapy and COVID-19. Annu Rev Immunol (2022) 40:323–48. doi: 10.1146/annurev-immunol-101220-023458 35113729

[78] Adriawan IR Atschekzei F Dittrich-Breiholz O Garantziotis P Hirsch S Risser LM. Novel Aspects of Regulatory T Cell Dysfunction as a Therapeutic Target in Giant Cell Arteritis. Ann Rheum Dis (2022) 81:124–31. doi: 10.1136/annrheumdis-2021-220955 PMC8762021 34583923

[79] Miyabe C Miyabe Y Strle K Kim ND Stone JH Luster AD. An Expanded Population of Pathogenic Regulatory T Cells in Giant Cell Arteritis is Abrogated by IL-6 Blockade Therapy. Ann Rheum Dis (2017) 76:898–905. doi: 10.1136/annrheumdis-2016-210070 27927642 PMC5744591

[80] Samson M Greigert H Ciudad M Gerard C Ghesquiere T Trad M. Improvement of Treg Immune Response After Treatment With Tocilizumab in Giant Cell Arteritis. Clin Transl Immunol (2021) 10:e1332. doi: 10.1002/cti2.1332 PMC8435365 34532040

[81] Stone JH Tuckwell K Dimonaco S Klearman M Aringer M Blockmans D. Trial of Tocilizumab in Giant-Cell Arteritis. N Engl J Med (2017) 377:317–28. doi: 10.1056/NEJMoa1613849 28745999

[82] Niederlova V Tsyklauri O Chadimova T Stepanek O. CD8(+) Tregs Revisited: A Heterogeneous Population With Different Phenotypes and Properties. Eur J Immunol (2021) 51:512–30. doi: 10.1002/eji.202048614 33501647

[83] Liu H Qiu F Wang Y Zeng Q Liu C Chen Y. CD8+CD122+PD-1+ Tregs Synergize With Costimulatory Blockade of CD40/CD154, But Not B7/CD28, to Prolong Murine Allograft Survival. Front Immunol (2019) 10:306. doi: 10.3389/fimmu.2019.00306 30863408 PMC6399415

[84] Menager-Marcq I Pomie C Romagnoli P Van Meerwijk JP. CD8+CD28- Regulatory T Lymphocytes Prevent Experimental Inflammatory Bowel Disease in Mice. Gastroenterology (2006) 131:1775–85. doi: 10.1053/j.gastro.2006.09.008 PMC1950262 17087950

[85] Wen Z Shimojima Y Shirai T Li Y Ju J Yang Z. NADPH Oxidase Deficiency Underlies Dysfunction of Aged CD8+ Tregs. J Clin Invest (2016) 126:1953–67. doi: 10.1172/JCI84181 PMC4855948 27088800

[86] Jin K Parreau S Warrington KJ Koster MJ Berry GJ Goronzy JJ. Regulatory T Cells in Autoimmune Vasculitis. Front Immunol (2022) 13:844300. doi: 10.3389/fimmu.2022.844300 35296082 PMC8918523

[87] Kong X Sun Y Ma L Chen H Wei L Wu W. The Critical Role of IL-6 in the Pathogenesis of Takayasu Arteritis. Clin Exp Rheumatol (2016) 34:S21–7. 26633132

[88] Jiang W Sun M Wang Y Zheng M Yuan Z Mai S. Notch1 Licenses mTOR Hyperactivity and Drives Vascular Inflammation in Patients With Takayasu's Arteritis. Arthritis Rheumatol (2022). doi: 10.1002/art.42103 35212196

[89] Maciejewski-Duval A Comarmond C Leroyer A Zaidan M Le Joncour A Desbois AC. mTOR Pathway Activation in Large Vessel Vasculitis. J Autoimmun (2018) 94:99–109. doi: 10.1016/j.jaut.2018.07.013 30061014

[90] Savioli B Salu BR De Brito MV Vilela Oliva ML De Souza AWS. Silent Arterial Inflammation During the Apparent Remission State of Takayasu's Arteritis. What do Cytokines Tell Us? Clin Exp Rheumatol (2018) 36 Suppl 111:33–9. 29600943

[91] Jia W Fu ZL Wang X Luo J Yan CL Cao JP. Decreased Absolute Number of Circulating Regulatory T Cells in Patients With Takayasu's Arteritis. Front Immunol (2021) 12:768244. doi: 10.3389/fimmu.2021.768244 35006213 PMC8732761

[92] Seko Y Minota S Kawasaki A Shinkai Y Maeda K Yagita H. Perforin-Secreting Killer Cell Infiltration and Expression of a 65-kD Heat-Shock Protein in Aortic Tissue of Patients With Takayasu's Arteritis. J Clin Invest (1994) 93:750–8. doi: 10.1172/JCI117029 PMC293919 7906697

[93] Matsumoto K Suzuki K Yoshimoto K Seki N Tsujimoto H Chiba K. Significant Association Between Clinical Characteristics and Changes in Peripheral Immuno-Phenotype in Large Vessel Vasculitis. Arthritis Res Ther (2019) 21:304. doi: 10.1186/s13075-019-2068-7 31888748 PMC6937853

[94] Langford CA Cuthbertson D Ytterberg SR Khalidi N Monach PA Carette S. A Randomized, Double-Blind Trial of Abatacept (CTLA-4Ig) for the Treatment of Giant Cell Arteritis. Arthritis Rheumatol (2017) 69:837–45. doi: 10.1002/art.40044 PMC5378642 28133925

[95] Langford CA Cuthbertson D Ytterberg SR Khalidi N Monach PA Carette S. A Randomized, Double-Blind Trial of Abatacept (CTLA-4Ig) for the Treatment of Takayasu Arteritis. Arthritis Rheumatol (2017) 69:846–53. doi: 10.1002/art.40037 PMC5378643 28133931

[96] Conway R O'neill L Gallagher P Mccarthy GM Murphy CC Veale DJ. Ustekinumab for Refractory Giant Cell Arteritis: A Prospective 52-Week Trial. Semin Arthritis Rheum (2018) 48:523–8. doi: 10.1016/j.semarthrit.2018.04.004 29776658

[97] Terao C Yoshifuji H Nakajima T Yukawa N Matsuda F Mimori T. Ustekinumab as a Therapeutic Option for Takayasu Arteritis: From Genetic Findings to Clinical Application. Scand J Rheumatol (2016) 45:80–2. doi: 10.3109/03009742.2015.1060521 26313121

[98] Gon Y Yoshifuji H Nakajima T Murakami K Nakashima R Ohmura K. Long-Term Outcomes of Refractory Takayasu Arteritis Patients Treated With Biologics Including Ustekinumab. Mod Rheumatol (2021) 31:678–83. doi: 10.1080/14397595.2020.1800560 32700985

[99] Matza MA Fernandes AD Stone JH Unizony SH. Ustekinumab for the Treatment of Giant Cell Arteritis. Arthritis Care Res (Hoboken) (2021) 73:893–7. doi: 10.1002/acr.24200 32248659

[100] Cid MC Unizony SH Blockmans D Brouwer E Dagna L Dasgupta B. Efficacy and Safety of Mavrilimumab in Giant Cell Arteritis: A Phase 2, Randomised, Double-Blind, Placebo-Controlled Trial. Ann Rheum Dis (2022) 81(5):653–61. doi: 10.1136/annrheumdis-2021-221865 PMC8995812 35264321

[101] Venhoff N Schmidt WA Lamprecht P Tony HP App C Sieder C. Efficacy and Safety of Secukinumab in Patients With Giant Cell Arteritis: Study Protocol for a Randomized, Parallel Group, Double-Blind, Placebo-Controlled Phase II Trial. Trials (2021) 22:543. doi: 10.1186/s13063-021-05520-1 34404463 PMC8369438

[102] Smolen JS Landewe RBM Bijlsma JWJ Burmester GR Dougados M Kerschbaumer A. EULAR Recommendations for the Management of Rheumatoid Arthritis With Synthetic and Biological Disease-Modifying Antirheumatic Drugs: 2019 Update. Ann Rheum Dis (2020) 79:685–99. doi: 10.1136/annrheumdis-2019-216655 31969328

[103] Watanabe R Hashimoto M. Perspectives of JAK Inhibitors for Large Vessel Vasculitis. Front Immunol (2022) 13:881705. doi: 10.3389/fimmu.2022.881705 35432355 PMC9005632

[104] Régnier P Le Joncour A Maciejewski-Duval A Desbois AC Comarmond C Rosenzwajg M. Targeting JAK/STAT Pathway in Takayasu's Arteritis. Ann Rheum Dis (2020) 79:951–9. doi: 10.1136/annrheumdis-2019-216900 32213496

[105] Vieira M Regnier P Maciejewski-Duval A Le Joncour A Darasse-Jeze G Rosenzwajg M. Interferon Signature in Giant Cell Arteritis Aortitis. J Autoimmun (2022) 127:102796. doi: 10.1016/j.jaut.2022.102796 35123212

[106] Watanabe R Berry GJ Liang DH Goronzy JJ Weyand CM. Cellular Signaling Pathways in Medium and Large Vessel Vasculitis. Front Immunol (2020) 11:587089. doi: 10.3389/fimmu.2020.587089 33072134 PMC7544845

[107] Terao C Yoshifuji H Kimura A Matsumura T Ohmura K Takahashi M. Two Susceptibility Loci to Takayasu Arteritis Reveal a Synergistic Role of the IL12B and HLA-B Regions in a Japanese Population. Am J Hum Genet (2013) 93:289–97. doi: 10.1016/j.ajhg.2013.05.024 PMC3738822 23830516

[108] Kadoba K Watanabe R Iwasaki T Nakajima T Kitagori K Akizuki S. A Susceptibility Locus in the IL12B But Not LILRA3 Region is Associated With Vascular Damage in Takayasu Arteritis. Sci Rep (2021) 11:13667. doi: 10.1038/s41598-021-93213-9 34211061 PMC8249518

[109] Watanabe R. JAK Inhibitors as Promising Agents for Refractory Takayasu Arteritis. Ann Rheum Dis (2022) 81:e67. doi: 10.1136/annrheumdis-2020-217577 32340977

